# Correlation of exercise participation, behavioral inhibition and activation systems, and depressive symptoms in college students

**DOI:** 10.1038/s41598-023-43765-9

**Published:** 2023-09-30

**Authors:** Shufan Li, Xing Wang, Peng Wang, Shali Qiu, Xin Xin, Jing Wang, Jinlei Zhao, Xiaojing Zhou

**Affiliations:** 1 Shanghai University of Sport, Shanghai, China; 2 School of Physical Education and Health, Shanghai Lixin University of Accounting and Finance, No.2800 Wenxiang Road, Songjiang District, Shanghai, China

**Keywords:** Psychology, Diseases

## Abstract

To clarify the pathways and effects of the behavioral inhibition and activation systems in the relationship between exercise participation and depressive symptoms among college students. A cross-sectional research design was employed to survey 2606 college students using physical activity questionnaires, the Behavioral Inhibition/Activation Systems Scale, and the Beck Depression Inventory. Data were analyzed using methods including one-way ANOVA, independent sample t-tests, non-parametric tests, chi-square tests, correlation analysis, and structural equation modeling. Depressive symptoms were significantly negatively correlated with exercise participation (*r* = − 0.107, *P* < 0.001), reward responsiveness (*r* = − 0.201, *P* < 0.001), drive (*r* = − 0.289, *P* < 0.001), and fun seeking (*r* = − 0.102, *P* < 0.001), and positively correlated with behavioral inhibition (*r* = 0.084, *P* < 0.001). Exercise participation was positively correlated with reward responsiveness (*r* = 0.067, *P* = 0.001), drive (*r* = 0.085, *P* < 0.001), and fun seeking (*r* = 0.063, *P* = 0.001). Exercise participation had a significant direct effect (B = − 0.079, 95% CI − 0.116 to − 0.043) and total effect (B = − 0.107, 95% CI − 0.148 to − 0.069) on depressive symptoms. The mediating effects of drive (B = − 0.028, 95% CI − 0.043 to − 0.016) and fun seeking (B = 0.005, 95% CI − 0.001 to 0.011) were significant. The more college students engage in exercise, the lower their depressive symptom scores. Drive and fun seeking mediate the relationship between college students' exercise participation and depressive symptoms. Encouraging exercise participation among college students and enhancing their sensitivity to behavioral activation strategies and reward information may have a significant role in preventing and alleviating depressive symptoms.

## Introduction

Approximately 450 million people globally suffer from mental health issues, with depression, anxiety, and stress being the leading psychological disorders^1^. More than 320 million individuals experience varying degrees of depressive symptoms^2^, which can manifest as persistent low mood, reduced interest, and lethargy, accompanied by cognitive, physiological, and behavioral disturbances^3,4^. In severe cases, individuals may even contemplate or engage in self-harm or suicide. In recent years, there has been a trend toward younger populations experiencing depression, with a total prevalence rate of 33.6% for depressive symptoms among college students^5^. Effective prevention and intervention strategies for depressive symptoms in college students are urgently needed.

Depressive symptoms are closely associated with the Behavioral Inhibition System (BIS) and Behavioral Activation System (BAS). BAS is highly sensitive to rewards, non-punishment, and avoidance of punishment stimuli, leading to approach behavior and facilitating positive emotional experiences. On the other hand, BIS is highly sensitive to punishment, non-reward, and novelty stimuli, resulting in withdrawal and avoidance behavior, inhibiting individual behavioral responses, and contributing to negative emotional experiences^6,7^. Research has indicated that BAS can serve as a predictive indicator of depression risk, with low BAS intensity being a stable marker of vulnerability to depressive symptoms^8,9^. PINTO-MEZA et al. found that depressive symptoms were associated with decreased BAS functioning^10^. However, some studies suggest that individuals with depressive symptoms may exhibit both decreased BAS functioning and enhanced BIS functioning^11^. Individuals with high behavioral activation are better equipped to cultivate positive emotions, easily recover from negative emotions, and possess the psychological resilience to manage daily stressors^12^.

There is a close association between exercise participation, the BIS, the BAS, and depressive symptoms. Exercise participation has been shown to enhance the BAS and improve depressive symptoms^13–15^. Studies have found that college students who engage in higher levels of exercise have lower rates of depressive symptoms, and effective physical activity can, to some extent, prevent the occurrence of depressive emotions^16–18^. Additionally, exercise can stimulate neurotransmitter release, promote the secretion and absorption of dopamine in the brain, thereby enhancing the individual's BAS, and fostering feelings of pleasure and happiness, contributing to improved mental health^19^. Research has also revealed a positive correlation between high exercise participation and BAS, while low exercise participation is positively correlated with BIS^20^. Individuals with high BAS exhibit greater motivation for engaging in physical activities and experience positive emotional responses to increased exercise^21^.

Reviewing previous literature, it is evident that exercise is closely related to depressive symptoms^13^, exercise can enhance the BAS, and both behavioral inhibition and activation systems are influencing factors of depressive symptoms^22^. However, previous research has not validated the relationship between exercise participation, behavioral inhibition and activation systems, and depressive symptoms within the college student population. Furthermore, the mediating role of the behavioral inhibition and activation systems in the relationship between exercise participation and depressive symptoms has not been clarified. By examining previous studies, it is clear that the following questions still need to be addressed: Is there a relationship between exercise participation, behavioral inhibition and activation systems, and depressive symptoms among college students? If there is a relationship, based on this triad, can the behavioral inhibition and activation systems mediate the relationship between exercise participation and depressive symptoms in college students, and which dimensions among the four sub-dimensions—behavioral inhibition system, reward responsiveness, drive, and fun seeking—mediate this relationship? Therefore, this study plans to use a cross-sectional design to address the aforementioned research questions, aiming to provide clinical insights and offer a theoretical foundation for researchers and university administrators.

## Research subjects and methods

### Research subjects

The research subjects are college students currently enrolled in universities, aged 18 to 22 years old. Sample size estimation was conducted using the Monte Carlo mediation effect statistical power analysis principle, and the pwrSEM software was utilized for this purpose (the website is: yilinandrewang.shinyapps.io/pwrSEM/). This process involved defining the model, visualization, setting parameter values, and estimating statistical power, among other steps. Effect sizes were set based on previous literature^23–25^, the significance level α was set at 0.05, and the number of simulation runs was set at 5000. When the sample size reached 2000, the statistical power for the mediation effect exceeded 0.8. Considering a potential 10% sample loss rate, it is planned to survey a total of 2200 individuals.

**Figure 1:**
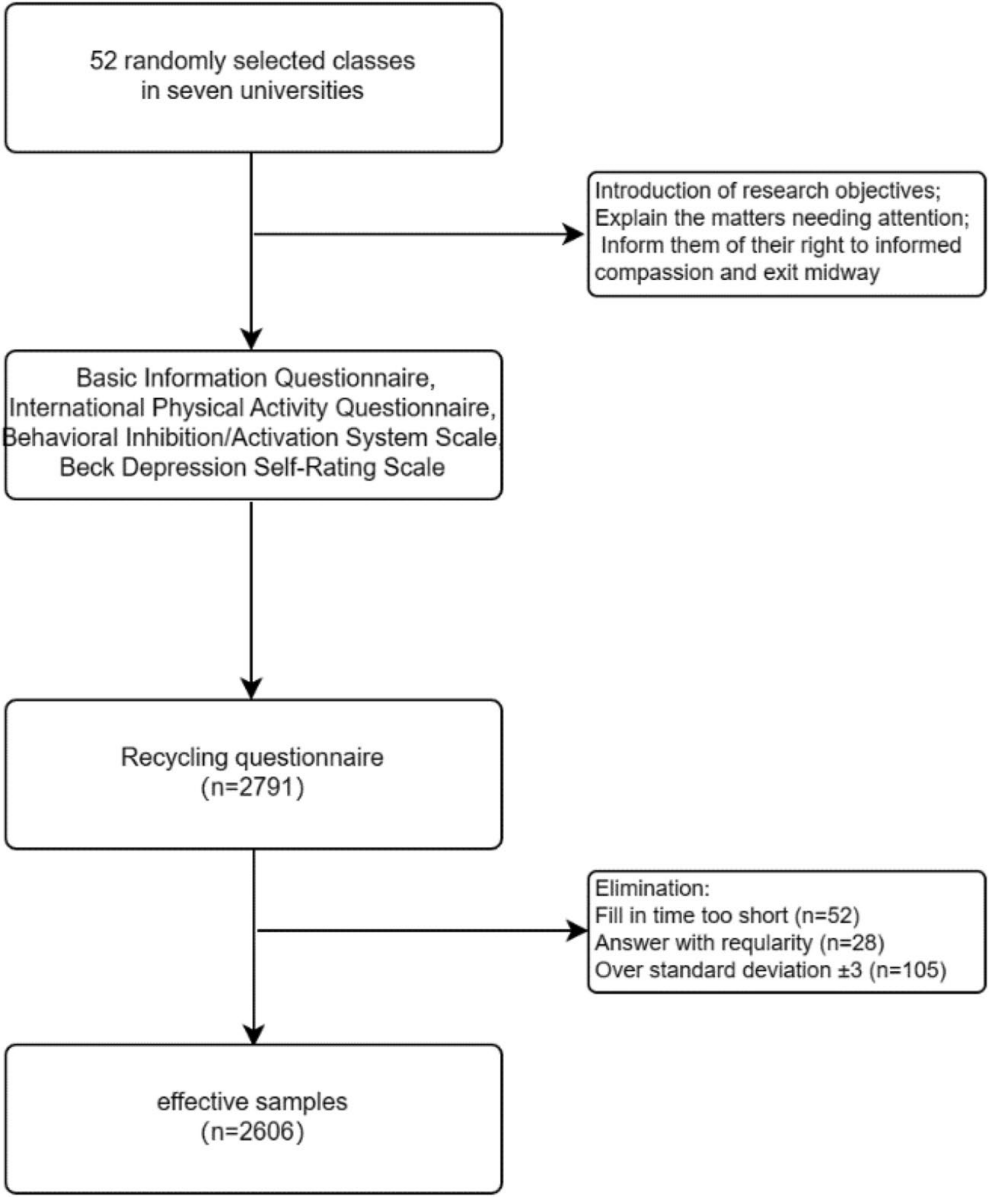
Flow chart for inclusion of samples.

In this study, a cluster sampling method was employed, and classes were randomly selected from seven universities in the Songjiang district of Shanghai. A total of 52 classes were selected, and 2791 college students were recruited to voluntarily participate in the study. They completed online questionnaires including basic information, a physical activity questionnaire, the Behavioral Inhibition/Activation Systems Scale, and the Beck Depression Inventory. Prior to completing the questionnaires, survey personnel read instructions and explained the items, clarifying that the data collected would be used solely for scientific research purposes. Participants were informed of the importance of providing truthful, independent, and voluntary responses, and they were assured of their right to withdraw from participation at any point. During the completion process, participants were reminded to answer carefully according to the instructions. After completion, survey personnel checked for missing responses and any content that contradicted common sense, ensuring data integrity, accuracy, and authenticity through follow-up and correction measures. A total of 185 invalid questionnaires were excluded, including 28 with systematically patterned responses, 52 with completion times less than 3 min, and 105 with responses exceeding ± 3 standard deviations. The study obtained 2606 valid questionnaires (93.37%). This study has been approved by the Ethics Committee of Shanghai University of Sport (approval number: 102772021RT004). The inclusion of the samples is shown in Fig. 1.

### Research tools

#### General information questionnaire

This questionnaire includes basic information about the research subjects, such as age, gender, height, weight, and family background.

#### International Physical Activity Questionnaire (IPAQ)

The IPAQ measures physical activity using metabolic equivalents (METs) as the standard. It assesses not only common forms of exercise but also takes into account activities like walking in daily life. The short form of the IPAQ, known as IPAQ-short, consists of seven questions. Six questions inquire about an individual's physical activity level, which is categorized into three intensity levels: high, moderate, and low. High-intensity physical activities include activities such as lifting heavy objects, digging, aerobic exercise, or fast cycling. Moderate-intensity activities include tasks like carrying light items, cycling at a normal pace, or playing doubles tennis. Low-intensity activities involve walking for at least 10 min at a time. Respondents are asked about the frequency of engaging in activities of different intensities over a week and the cumulative time spent each day. The MET values for high, moderate, and low intensities are 8.0, 4.0, and 3.3, respectively. The physical activity level (MET-min/week) for each intensity is calculated as the corresponding MET value multiplied by the number of days per week and the duration of activity per day. The overall physical activity level is obtained by summing the values for the three intensity levels. The retest reliability coefficient for IPAQ-short in this study was 0.718^26^.

#### Behavioral Inhibition/Activation System Scale (BIS/BAS Scale)

The BIS/BAS Scale was developed by Carver and White in 1994^27^. It divides the scale into two dimensions: BAS and BIS. In this study, the scale has been modified into four levels^28^, including BIS and three sub-dimensions of BAS: Reward Responsiveness, Drive, and Fun Seeking, totaling 18 items. BIS consists of items 5, 9, 18, 12, and 14, while BAS includes three subfactors: Reward Responsiveness (items 2, 4, 17, 13), Drive (items 1, 16, 6, 8), and Fun Seeking (items 7, 11, 15, 10, 3). Responses are collected using a 4-point Likert scale, ranging from "completely agree" to "completely disagree," with scores ranging from 1 to 4 for each item. The Cronbach's α coefficient for this scale in this study was 0.759^28^.

#### Beck Depression Inventory-II (BDI-II)

The BDI-II is used to assess an individual's depressive symptoms and has good reliability and validity^29^. The inventory categorizes depression into three dimensions: (1) Negative Attitudes or Suicidal Thoughts, which includes pessimism and feelings of helplessness; (2) Physical Symptoms, which encompasses fatigue and sleep disturbances; and (3) Difficulty in Functioning, which involves a perceived increase in the difficulty of performing tasks. The inventory comprises 21 items, and responses are scored on a scale of 0 to 3, with "0" indicating the absence of the symptom, "1" indicating mild, "2" indicating moderate, and "3" indicating severe. Total scores ranging from 0 to 13 indicate no depression, 14 to 19 indicate mild depression, 20 to 28 indicate moderate depression, and 29 to 63 indicate severe depression. The internal consistency coefficient for the BDI-II in this study was 0.948^30^.

### Data processing

Data were analyzed using SPSS 26.0 software. For group comparisons of continuous variables, independent sample t-tests were employed. For significantly skewed continuous data, the Mann–Whitney U test was used for group comparisons. In cases where questionnaire data were missing non-randomly to avoid bias in coefficient estimation due to the use of listwise deletion, similar mean imputation was applied. Frequency histograms were used to observe data distributions. Parametric tests were used for metric data that followed a normal or approximate normal distribution, and non-parametric tests were used for non-normally distributed data. Count data were described as n (%), and group comparisons were conducted using the *χ*^2^ test. Pearson correlation analysis was used to explore the relationships between exercise participation, depressive symptoms, and the Behavioral Inhibition and Activation Systems.

To detect common method bias, the Harman single-factor test was applied. Structural equation modeling was conducted using Amos 23.0 to examine the mediating role of the Behavioral Inhibition and Activation Systems in the relationship between exercise participation and depressive symptoms (all variables were standardized prior to modeling). Path analysis parameter estimation was performed using the non-parametric percentile bootstrap method (without strict assumptions about variable distributions). A total of 5000 samples were drawn, and statistical significance for the mediating effect was defined as the bias-corrected 95% confidence interval (Bias-Corrected 95% CI) not including 0. All statistical inferences were conducted using two-tailed tests, with a significance level of α set at 0.05, where *P* < 0.05, *P* < 0.01, and *P* < 0.001 indicated statistical significance.

### Ethics approval and consent to participate

For experiments involving human participants, informed consent has been obtained from all subjects (all adults) in this study. Our study was approved by the ethical committee of Shanghai University of Sport (102772021RT007). All methods were carried out in accordance with relevant guidelines and regulations.

## Results

### Differences in demographic variables, exercise participation, and behavioral inhibition and activation systems among university students with different depression scores

As shown in Table 1, a total of 2606 university students participated in the study, with an average age of (19.31 ± 1.428) years and a BMI of (21.494 ± 3.103) kg/m^2^. Male students accounted for 66.77% of the sample, while 52.99% were only children, and 9.40% came from single-parent families. Their average exercise participation was 1384 ± 1181 MET-min/week, and 357 students (13.70% of the total sample) exhibited depressive symptoms. Statistical significance was observed in differences between university students with depressive symptoms and those without in terms of single-parent families, exercise participation, and the Behavioral Inhibition and Activation Systems (*P* < 0.05), while no significant differences were found in other demographic variables (*P* > 0.05).

**Table 1:** Comparison of differences in demographic variables, exercise participation, and behavioral inhibition and activation systems among university students with different depression scores.

Variables	Depressive symptoms score			Test of variability
	Whole (2606)	Depression (357)	Normal (2249)	
Age/year	19.31 ± 1.428	19.41 ± 1.403	19.30 ± 1.431	*t* = 1.382, *P* = 0.167
BMI (kg/m^2^)	21.494 ± 3.103	21.595 ± 3.334	21.478 ± 3.065	*t* = 0.623, *P* = 0.533
Sex (male/%)	66.77%	63.31%	32.68%	*χ*^*2*^ = 2.237, *P* = 0.135
Only child (yes/%)	52.99%	52.94%	53%	*χ*^*2*^ < 0.001, *P* = 0.983
Single-parent family (yes/%)	9.40%	12.61%	8.89%	*χ*^*2*^ = 4.985, *P* = 0.026
Physical activity (MET-min/week)	1384 ± 1181	1160 ± 1140	1420 ± 1184	*Z* = 14.691, *P* < 0.001
Reward responsiveness	12.80 ± 2.005	11.95 ± 2.00	12.93 ± 1.973	*t* = − 8.601, *P* < 0.001
Drive	12.43 ± 2.100	11.24 ± 2.005	12.62 ± 2.053	*t* = − 11.830, *P* < 0.001
Fun seeking	14.71 ± 2.322	14.29 ± 2.172	14.77 ± 2.339	*t* = − 3.655, *P* < 0.001
Behavioral inhibition	14.29 ± 2.581	14.62 ± 2.411	14.24 ± 2.604	*t* = 2.584, *P* < 0.05

*Note* BMI, Body Mass Index; kg/m^2^, kilograms per square meter; Only child, refers to a child who is the sole offspring of a couple; MET-min/week, metabolic equivalents-min/week.

### Relationship between exercise participation, behavioral inhibition and activation systems, and depression symptoms in university students

**Figure 2:**
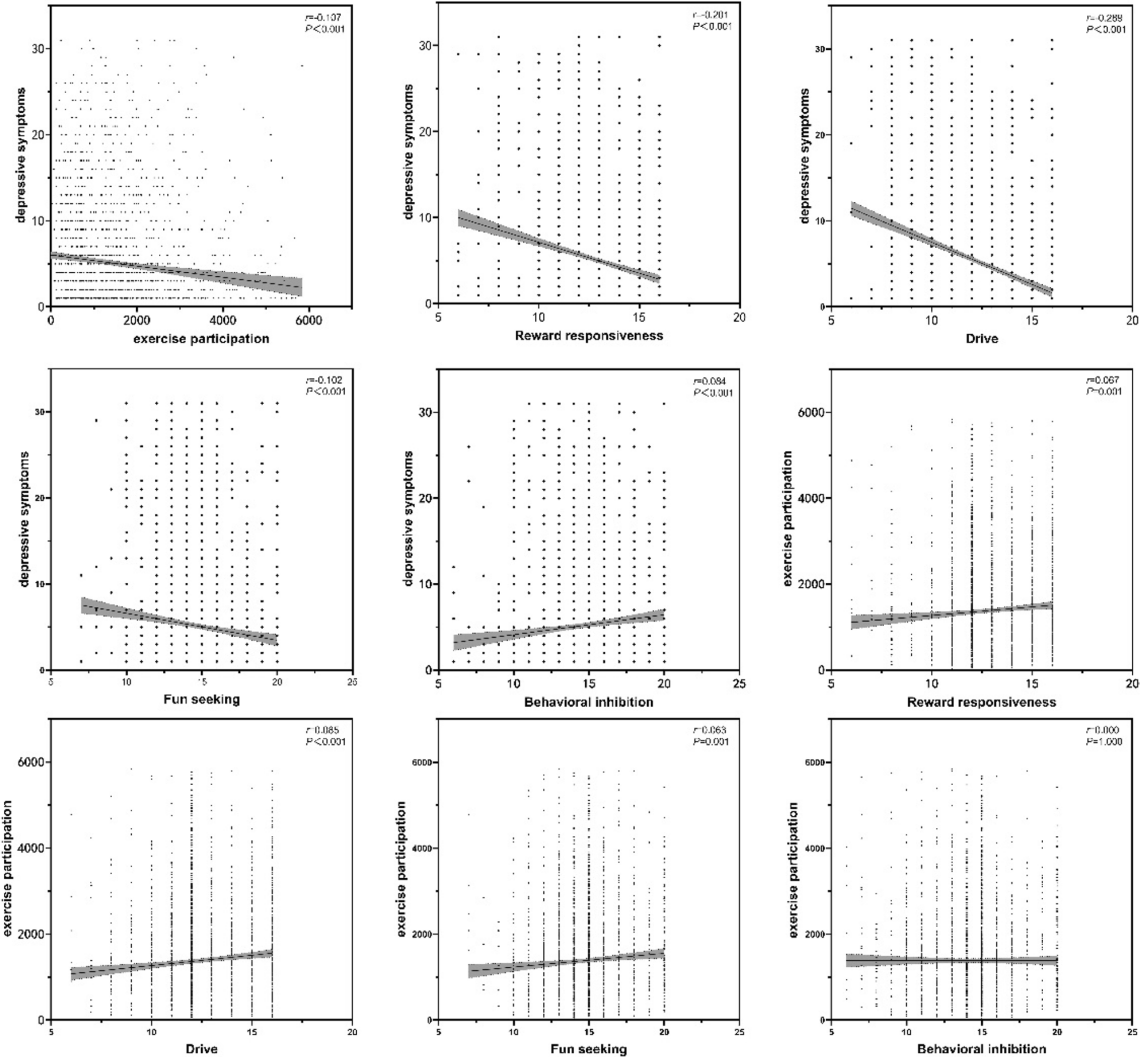
Relationship between exercise participation, behavioral inhibition and activation systems, and depression symptoms in university students.

The relationship between depression symptoms, exercise participation, and behavioral inhibition and activation systems was examined using Pearson correlation coefficients. The results (Fig. 2) showed a significant negative correlation between depression symptoms and exercise participation (*r* = − 0.107, *P* < 0.001), reward responsiveness (*r* = − 0.201, *P* < 0.001), drive (*r* = − 0.289, *P* < 0.001), and fun seeking (*r* = − 0.102, *P* < 0.001). Additionally, there was a significant positive correlation between depression symptoms and behavioral inhibition (*r* = 0.084, *P* < 0.001). On the other hand, exercise participation exhibited significant positive correlations with reward responsiveness (*r* = 0.067, *P* = 0.001), drive (*r* = 0.085, *P* < 0.001), and fun seeking (*r* = 0.063, *P* = 0.001), but it was unrelated to behavioral inhibition (*r* = 0.000,* P* = 1.000). These results suggest that increased exercise participation in university students is associated with lower depression symptom scores and higher behavioral activation system scores.

### Construction and validation of the structural model of college student exercise participation, depression symptoms, and behavioral inhibition and activation systems

To address the potential issue of common method bias in this study, a Harman's single-factor test was conducted. The results showed that there were nine eigenvalues greater than 1, and the variance explained by the first common factor was 23.838%, which was below the critical threshold of 40%. Therefore, there was no significant common method bias in this study.

Based on the relationships among college student exercise participation, depression symptoms, and behavioral inhibition and activation systems, a structural model was established. Exercise participation was treated as the independent variable, depression symptoms as the dependent variable, and behavioral inhibition and activation systems as the mediating variable. The model was refined by sequentially removing non-significant mediation paths and re-calculating until all mediation path coefficients were statistically significant according to bootstrap testing. The fit indices of the structural equation model were as follows: CMIN/df < 0.001, RMR < 0.001, RMSEA < 0.001, GFI = 1.000, NFI = 1.000, CFI = 1.000. These fit indices met the reference standards, with CMIN/df < 3, RMR < 0.05, RMSEA < 0.08, GFI, NFI, and CFI > 0.9^25^, indicating a good fit for the structural equation model and its reliability.

**Figure 3:**
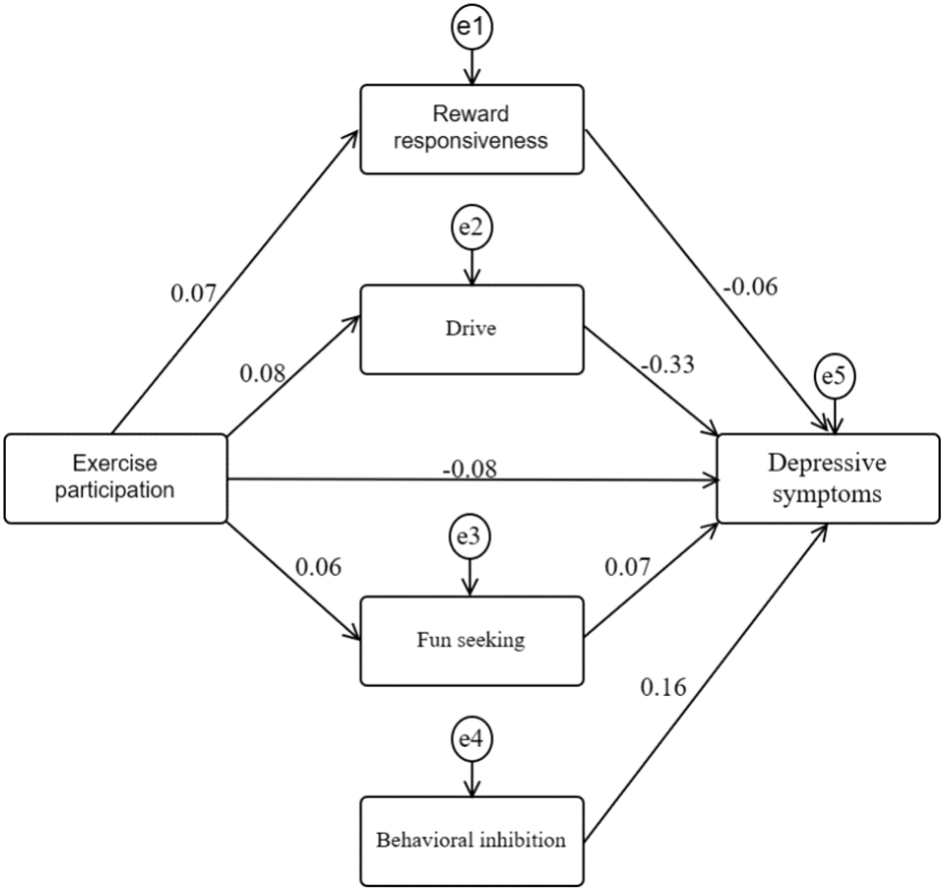
Mediating effects of exercise participation, behavioral inhibition and activation systems, and depression symptoms.

The path analysis is presented in Fig. 3, and the results of the mediation analysis are shown in Table 2. The path from exercise participation to behavioral inhibition was found to be non-significant and was removed (standardized regression coefficient β = 0, *P* = 1). The mediating effect through reward responsiveness was not significant, as the 95% confidence interval for this path included 0. However, the mediating effects through drive and fun seeking were significant. Exercise participation had significant direct and total effects on depression symptoms. Therefore, it can be concluded that the behavioral activation system can mediate the relationship between college student exercise participation and depression symptoms, primarily through the mediation of drive and fun seeking.

**Table 2:** List of intermediary effect coefficients.

Types of effects	B	SE	Bias-corrected 95% CI		*P*
The mediating effect of reward responsiveness	− 0.004	0.002	− 0.011	0	0.035
The mediating effect of the drive	− 0.028	0.007	− 0.043	− 0.016	0.001
The mediating effect of fun seeking	0.005	0.002	0.001	0.011	0.006
Direct effect	− 0.079	0.018	− 0.116	− 0.043	0.001
Total effect	− 0.107	0.02	− 0.148	− 0.069	0.001

*Note* B, path coefficient; SE, standard error; Bias-Corrected 95% CI, Bias-corrected 95% confidence interval.

## Discussion

The results of this study indicate that the more college students engage in physical activity, the lower their depression scores. Additionally, exercise participation has a direct effect on reducing depression symptoms, which is consistent with previous research. Wang et al.^31^ found that physical activity behavior among adolescents has a direct effect of 39.7% on their depression symptoms. The intensity, duration, and frequency of physical activity among adolescents are all moderately negatively correlated with depression symptoms. Increased physical activity has a protective effect against depression, with longer durations of physical activity associated with lower depression rates^32^. Exercise can improve various manifestations of depression symptoms. Exercise can elevate endorphin levels, help regulate stress-related body temperature and cardiovascular systems, enhance mood, and alleviate pain. β-endorphins in the brain, in particular, play a role in preventing the compulsive inhibitory effect of dopamine (a major neurotransmitter in brain regions related to pleasure and motivation, such as the ventral tegmental area and nucleus accumbens), thus positively influencing mood^33^. Exercise can also increase cerebral blood flow, enhance the volume of certain brain regions like the prefrontal cortex, and maintain the integrity of white matter^34^. Furthermore, exercise can promote the expression of brain-derived neurotrophic factor (BDNF), facilitating neuronal growth, survival, synaptogenesis, and repair, which effectively improves cognitive function^35^. In addition to these benefits, individuals with depression often experience sleep disturbances. The level of physical activity is negatively correlated with the severity of sleep disturbances among this population^36^, improving various sleep outcomes in adults, such as total sleep time, sleep efficiency, and sleep quality^37^.

Our study found that the BAS can mediate the relationship between college students' physical activity and depression symptoms through two pathways: "drive" and "fun seeking." One possible neurobiological mechanism is that the BAS is located within the midbrain dopamine circuitry. Exercise promotes the release of neurotransmitters such as dopamine, serotonin, endorphins, and norepinephrine, which regulate mood. This increase in dopamine levels may enhance the ventral tegmental area-nucleus accumbens pathway, positively affecting mood^38,39^.

The "drive" pathway may mediate the relationship between exercise and depression symptoms. "Drive" refers to persistent behavior in pursuit of a goal. There is an inverted U-shaped relationship between "drive" and physiological activation levels, where moderate "drive" induces the optimal level of physiological activation^40^. Research has shown that physical activity can moderately increase an individual's "drive"^20^. "Drive" is a good predictor of treatment outcomes and is beneficial for the social functioning recovery of individuals with depression symptoms^41^. Moreover, "drive" has a direct impact on non-suicidal self-injury behavior, as individuals with highly sensitive "drive" have a strong inclination to move toward their goals, even in maladaptive ways. This strong inclination may drive individuals to engage in self-injurious behavior to achieve short-term benefits, such as regulating emotions, even in the context of negative emotions^42^.

The "fun seeking" pathway may also mediate the relationship between exercise and depression symptoms. "Fun seeking" refers to an individual's tendency to seek stimulation and potential rewarding situations. Moderate "fun seeking" increases sensitivity to rewarding information in the environment and promotes participation in social activities. "Fun seeking" is a factor influencing depression symptoms and is a risk factor for non-suicidal self-injury behavior^43^. Individuals with high sensitivity to "fun seeking" have high expectations for predictable rewards and may experience intense negative emotions if their desires are not fulfilled, leading to impulsivity and self-injurious behavior to obtain short-term benefits, such as emotional regulation^44–46^. Therefore, moderate "drive" and "fun seeking" may have a positive effect on the prevention and alleviation of depression symptoms.

This study has several limitations. Firstly, it adopts a cross-sectional design, relying solely on subjective reports, which may introduce certain biases. Future research should employ longitudinal approaches and objective assessment tools to further substantiate these findings. Secondly, the study does not investigate whether "drive" and "fun seeking" are influenced by other moderating variables when acting as mediators. Future research could explore the effects of moderating variables in greater depth. Lastly, the study does not identify the optimal points at which "drive" and "fun seeking" provide the most significant benefits. Future longitudinal studies should delve into this aspect more comprehensively.

## Conclusion

The more college students engage in exercise, the lower their depressive symptom scores. Drive and fun seeking mediate the relationship between college students' exercise participation and depressive symptoms. Encouraging exercise participation among college students and enhancing their sensitivity to behavioral activation strategies and reward information may have a significant role in preventing and alleviating depressive symptoms.

## Data Availability

The datasets used and/or analyzed during the current study are available from the corresponding author on reasonable request.
